# Death Caused by Amylene

**Published:** 1857-08

**Authors:** 


### Death Caused By Amylene.

Translated from L' Art Dentaire by CHAS. S. D'ARCAMBAL,
Pharmaceutical Chemist, Keokuk.

The discovery of a new anesthetic agent and its great efficacy without danger, also the facility of administering, is a subject that occupies, at this time, the study of many distinguished writers.

This question, so interesting to all practitioners, is of particular interest to dentists, from the fact that the problem so long sought, would be solved, viz:—the removal of teeth without pain.

Local anesthetics have given good results, but they are not always applicable, and the liability of deaths by the use of chloroform demands the greatest judgment when required to be employed in operations on the teeth. These considerations have no doubt already presented themselves to our distinguished lecturers, who are now turning their attention to Amylene, the new anesthetic agent, which engrosses the attention of the medical world at this moment. We are ourselves watching the progress of this interesting question, awaiting patiently the chemical and experimental studies to solve the same prior to presenting the facts to our readers. But a case has just transpired of too grave a nature to be passed over in silence by us. The medical journals have just published a case of death produced by Amylene. Without pretending to judge of this new anesthetic agent, which does not appertain to us, we believe it our duty to notify practitioners—who may have been induced to try the Amylene for the extracting of teeth, owing to so many favorable reports, as preferable to chloroform—of the following case, which will shows that Amylene cannot as yet be regarded as a substance to be employed without danger.

We copy from the Gazette des Hopsitaux, in relation to a case by M. Snow, who states it to be the first case that he has observed after a series of 143 favorable results with the anesthetic agent, which he introduced the employment of in England.

M. Ferguson requested me on the 7th of this month to assist him in an operation of fistula, which he was about to perform on a man aged 33 years, enjoying ordinary good health who complained of nothing but a local pain in his fistula.

Prior to the operation auscultation denoted sounds of the heart in their normal state; the pulse was natural, only a little excited at the moment of inhalation, but that vivacity was similar to those we observed prior to an operation. The patient lying on his side; I poured 6 drachms of Amylene in my apparatus (I never use all the liquid I pour out, because I wish merely to keep a dampness of the paper) and the patient commenced large and frequent inhalations. I advanced gradually the slide that closed the mask I use for inhalation, so that three-fourths of the opening was covered.

At the expiration of two minutes the patient was not conscious of anything that was taking place about him; his breathing was more frequent. Mr. Ferguson felt the pulse of the patient; it was good. I looked at my watch: it was two and a half minutes since he commenced inhalation; the patient was insensible; the operator sounded the track of the fistula; he took the bistoury and commenced the operation.

I held one leg of the patient; his limbs were rigid; I kept my eyes fixed upon him as much as possible while assisting in the operation. The cap which closed the inhaler was entirely closed. I was not paying much attention; I had closed the cap often, but what took place at this moment? The patient—did he draw a long breath? I know not. I suspended the inhalation because the simple incision that was necessary, being performed, the operation was ended.

I felt the patient's pulse half a minute before; it was good; and now, notwithstanding in the left there was no pulsation, on the right I could perceive a slight undulation.

His respiration was calm, and the patient, as if recovering consciousness, was moving his limbs, indicating his awaking. I watched with anxiety the patient's face, hoping that his respiration would re-establish his pulse. Two or three minutes elapsed; insensibility increased; the pupils of his eyes were slightly dilated, without provoking any winking; respiration was short and embarrassing.

The patient was livid, respiration anxious, the respiratory movements becoming less and less frequent; the respirations rare and deep. We thought of having recourse to the artificial method of respiration, of Dr. Marshall Hall.

After having placed him in a slightly elevated position, and opening his mouth, we exercised alternate pressure upon the thorax. During this time we distinctly heard the passage of air through the larynx. Insufflation, mouth to mouth, gave no further service. I heard a feeble beating at the throat; Mr. Ferguson felt slight pulsation at the right wrist.

We continued artificial respiration, but the patient showed no signs of life. The respiration continued after the cessation of the heart's action. At ten minutes before five pulsation was imperceptible, and at five o'clock the patient yet breathed. He had taken no nourishment for several hours, but had drank a pint of ale a little prior to the operation. A large amount of Amylene was still in the apparatus, after an hour and a half exposure to the air.

AUTOPSY 48 HOURS AFTER DEATH.

Body rigid; the integuments were raised up by a large quantity of fat. The cartilages of the ribs were ossified. The lungs were swelled but were not depressed; they filled completely the thoracic cavity and seemed emphysematous, although their surface did not present any large cells.

The posterior surface of the left lung was a little congested; the balance was not very vascular. The pericardium enclosed a small quantity of clear liquid. The heart was a little more enlarged than usual, offering a large quantity of fat on the surface.

On raising and cutting the large vessels three or four ounces of black blood escaped; slight dilation of the right ventricle. The septum of the left ventricle was thickened and so contracted as to close the cavity.

The liver was vascular, black and friable. The stomach was normal, containing a little mucus; the other organs were not examined. The body did not exhale, in the least, the odor of the Amylene.

Note again the fact, exceedingly curious, the persistence of respiration after paralysis of the heart.

